# Supplementary material for: Splice-Junction-Based Mapping of Alternative Isoforms in the Human Proteome
Source: Cell Rep. Author manuscript; Available in PMC 2020 Jan 15. (PMC6961840; doi:10.1016/j.celrep.2019.11.026)
Supplement: 2 [file NIHMS1546469-supplement-2.zip › DF1/PXD009737/TestisGluC/TestisGluC_11_LZTR1_RQIGAAALAGGARSKVAPSVD.pdf]

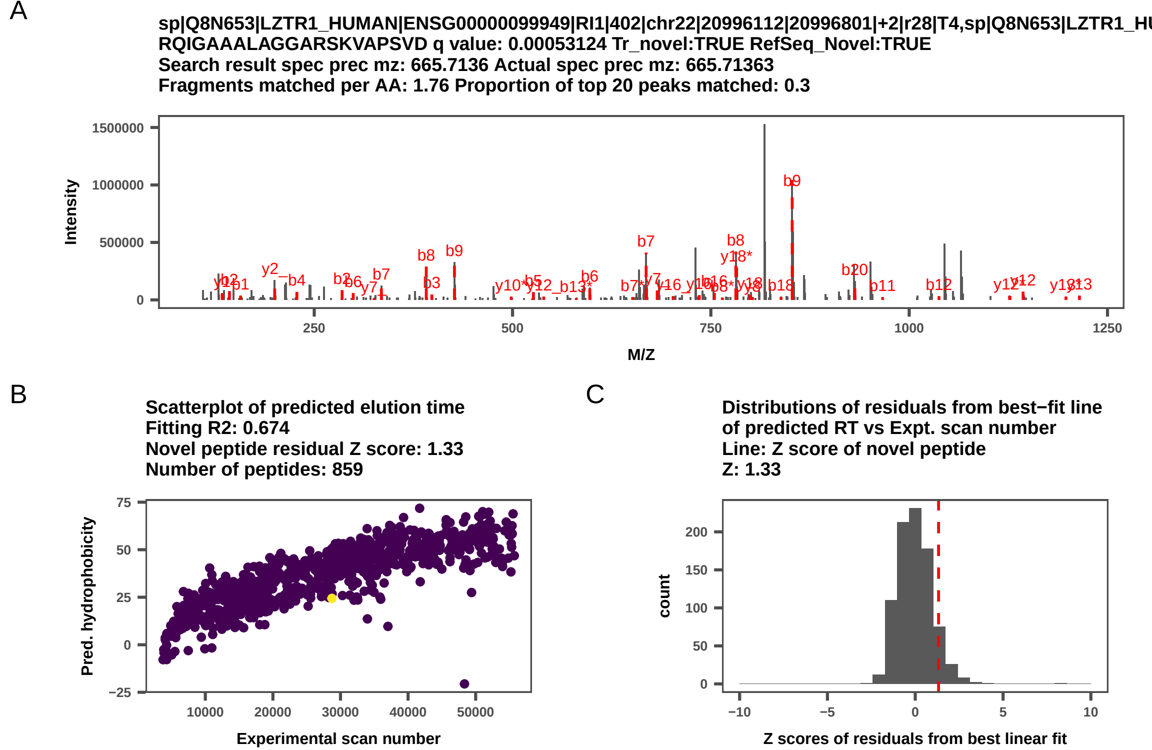
A
sp|Q8N653|LZTR1_HUMAN|ENSG00000099949|RI1|402|chr22|20996112|20996801|+2|r28|T4,sp|Q8N653|LZTR1_HU
RQIGAAALAGGARSKVAPSVD q value: 0.00053124 Tr_novel:TRUE RefSeq_Novel:TRUE
Search result spec prec mz: 665.7136 Actual spec prec mz: 665.71363
Fragments matched per AA: 1.76 Proportion of top 20 peaks matched: 0.3
Intensity
1500000
1000000
500000
0
M/Z
250
500
750
1000
1250
b9
b8
b9
b7
b8
y18*
b20
y2
b4
b7
b3
b6
b11
b12
b18
B
Scatterplot of predicted elution time
Fitting R2: 0.674
Novel peptide residual Z score: 1.33
Number of peptides: 859
Pred. hydrophobicity
75
50
25
0
−25
10000
20000
30000
40000
50000
Experimental scan number
C
Distributions of residuals from best−fit line
of predicted RT vs Expt. scan number
Line: Z score of novel peptide
Z: 1.33
count
200
150
100
50
0
−10
−5
0
5
10
Z scores of residuals from best linear fit
